# Author Correction: Women’s representation in Indian academia and conferences

**DOI:** 10.1038/s42003-024-06145-1

**Published:** 2024-04-18

**Authors:** Shruti Muralidhar, Vaishnavi Ananthanarayanan

**Affiliations:** EMBL Australia Node in Single Molecule Science, Department of Molecular Medicine, School of Biomedical Sciences, University of New South Wales, Sydney, Australia

**Keywords:** Careers, Institutions

Correction to: *Communications Biology* 10.1038/s42003-024-06058-z, published online 30 March 2024

The original version of the Article contained a duplicated Figure 6 in place of Figure 5.
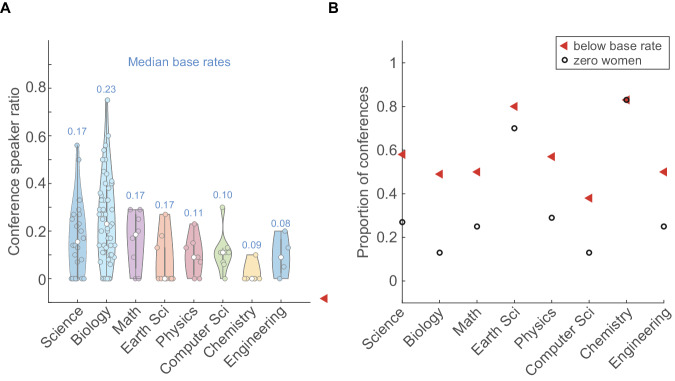


This has now been corrected in the PDF and HTML versions of the Article.

